# Initiative, Personality and Leadership in Pairs of Foraging Fish

**DOI:** 10.1371/journal.pone.0036606

**Published:** 2012-05-02

**Authors:** Shinnosuke Nakayama, Jennifer L. Harcourt, Rufus A. Johnstone, Andrea Manica

**Affiliations:** Department of Zoology, University of Cambridge, Cambridge, United Kingdom; Cajal Institute, Consejo Superior de Investigaciones Científicas, Spain

## Abstract

Studies of coordinated movement have found that, in many animal species, bolder individuals are more likely to initiate movement and shyer individuals to follow. Here, we show that in pairs of foraging stickleback fish, leadership is not merely a passive consequence of temperamental differences. Instead, the act of initiating a joint foraging trip out of cover itself brings about a change in the role that an individual plays throughout the subsequent trip, and success in recruiting a partner affects an individual's tendency to initiate the next trip. On each joint trip, whichever fish took the initiative in leading out of cover gains greater influence over its partner's behaviour, which persists even after several changes in position (i.e. termination attempts and re-joining). During any given trip, the initiator is less responsive to its partner's movements than during trips initiated by the partner. An individual's personality had an important effect on its response to failure to recruit a partner: while bold fish were unaffected by failures to initiate a joint trip, shy individuals were less likely to attempt another initiation after a failure. This difference provides a positive feedback mechanism that can partially stabilise social roles within the pair, but it is not strong enough to prevent occasional swaps, with individuals dynamically adjusting their responses to one another as they exchange roles.

## Introduction

Studies of leadership have shown that within animal groups, some individuals are consistently more likely than others to initiate and direct collective movement. Most recent work has focused on the characteristics of leaders, identifying the traits that distinguish these influential individuals from the rest of the group – those more likely to lead may be dominant [1], [2], better informed [3]–[5], hungrier [6]–[8], or simply temperamentally less inclined to follow others [9], [10]. Regardless of the particular characteristics in question, the assumption is that leadership is the outcome of pre-existing differences among individuals in how they tend to behave and react to one another.

In this paper, we focus not only on the characteristics of leaders, but also on the consequences of their actions. Specifically, we ask whether an individual that initiates coordinated group movement gains influence thereby, and alters the way in which others respond to it for the duration of that group movement. We also test whether leadership status during the current group movement influences initiation of the next group movement. If so, leadership is not just a passive outcome of pre-existing differences in temperament or physiological state. Instead, the act of successfully taking the lead itself brings about a change in the role that an individual plays within the group, both in the present and in the future. When individuals occasionally change roles between trips, their behaviour should then alter accordingly, and this effect should be observable over and above any persistent differences in behaviour attributable to temperament or physiological state.

To test this possibility, we use continuous-time Markov models to infer the rules underlying joint behaviour in pairs of foraging stickleback fish. In previous work [9], we showed that individuals in a pair of foraging stickleback fish respond to each other's movements based on their personality. Overall, the bolder individual was more likely to initiate joint trips out of cover, but that there were occasional changes in who initiated and who followed from one joint trip to the next. Here, we extend the Markov approach to assess whether and how the members of a pair alter their behaviour, and the ways in which they respond to one another, depending on which individual initiated the current joint trip out of cover. We also consider whether successful initiation of joint trips affects an individual's propensity to initiate in the future.

## Materials and Methods

### Acclimation and Training

**Figure 1:**
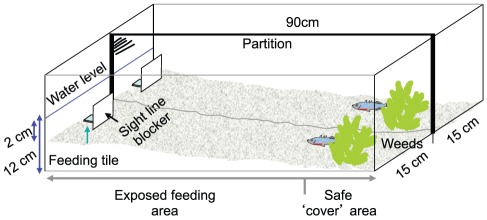
Diagrammatic represenation of the experimental set-up.

Procedures of animal collection and maintenance are found in Appendix S1. The experiments described below are the same as reported in Harcourt *et al.* [9]. All experiments were run in larger experimental tanks (90×30×30 cm; Figure 1), with walls covered by white plastic sheets and the bottom lined with gravel to create a slope (12 cm water depth at one end, 2 cm at the other end). Each tank was divided lengthwise into two lanes with a white opaque partition, and each lane contained two plastic plants at the deep end and one white square plastic plate (about 1.5 cm^2^) at the shallow end as a feeding site. A white plastic sheet (8×8 cm) was put vertically in front of each feeding plate to prevent fish at the deep end from seeing food on the plate at the shallow end. Prior to the experiments, we ran three one-hour-long training sessions, to acclimate the fish and train them to expect food at the feeding site. At the beginning of each session, a single medium-sized bloodworm was placed on the feeding plate, and fish were introduced individually at the deep end of each lane. After 30 min, an observer checked the food on the feeding plate, and a second bloodworm was placed on the feeding plate if the first had been consumed. At the end of each session, fish were returned to their individual holding tanks. For fish that failed to consume all the allotted food, the remainder was placed on the feeding plate in the holding tanks to ensure equal feeding among individuals.

### Assessment of Individual Temperament

After the training sessions, we ran two one-hour-long sessions during which no food was provided in the experimental tank, in order to assess the temperament of each fish. Following a 5-minute acclimation, fish behaviour was recorded from the top of the tank with a digital video camera (Sony SCR-35E, Sony, Japan). After recording, individuals were returned to their holding tanks and fed one bloodworm daily. Fish that failed to consume any food during the training sessions were excluded from the temperament assessments.

While watching the videos (speeded up at 4× normal speed), we recorded the times at which each individual left or returned to cover using a custom-made data logger. The time series of location transitions for each individual (from cover to exposed area, and vice-versa) was modelled as a continuous-time Markov chain with two states, either under cover or exposed (using msm v0.9.7 by Christopher Jackson in R2.11.1 by the R Core Development Team, as used in [11], [12]). Using this approach, we can estimate the transition intensities between the two locations, providing a measure of the tendency of a fish to leave and return cover. Individual temperament was defined as the ratio of the transition intensity for leaving cover to the transition intensity for returning to cover (higher temperament scores indicate “bolder" behaviour). This measure has been used in other studies as a reliable indicator of temperament [11], [13].

### Collective Movements in Pairs

After one additional training session, during which fish were again rewarded with food at the exposed end of the experimental tank, 40 individuals were haphazardly paired (n = 20 pairs). The movements of each pair were then video-recorded in the experimental tank twice with an opaque partition and twice with a transparent partition (through which the fish could observe each other and interact) for one hour each over four days. Individuals were returned to their individual holding tanks after each experiment. No food was provided during this period of observation, so as to eliminate any influence of changes in physiological state on an individual's social role as leader or follower [8], [14].

The timings of leaving and returning to cover were again recorded for each individual from the videos. For the experiments with an opaque partition, locations of individuals were categorized as either under cover or exposed, and individual temperament scores were calculated from the transition intensities between the two states in a continuous-time Markov model as described above. The temperament scores from this experiment were used for the later analyses, as they were highly correlated with those estimated during the previous week (*r* = 0.61, n = 40, *P*<0.001).

**Figure 2:**
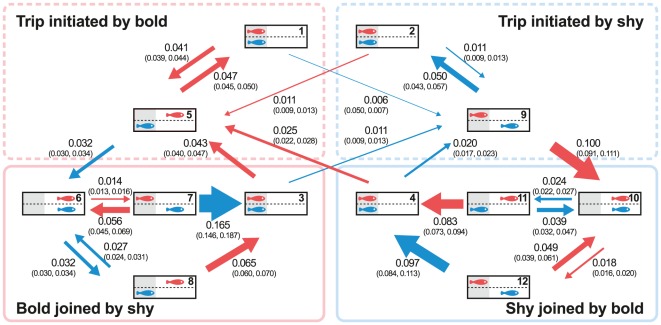
Transition intensities from the Markov Chain model. Transition intensities (best estimates and 95% CI) for leaving and returning to cover, estimated for the bold (red) and shy fish (blue) depending on which individual initiated the trip out of cover and whether it was successful in recruiting its partner. The width of each arrow is proportional to the magnitude of the transition intensity. For each state, the area under cover is shaded, while the exposed area is in white; each state is identified with a number at the top-right corner of the “tank".

In the analysis of Harcourt *et al.* [9], for the experiment with a transparent partition, the location of each individual at any given moment was categorised as either covered or exposed, yielding four possible states for a pair: bold fish exposed and shy fish covered, bold fish covered and shy fish exposed, both exposed and both covered. The transition intensities estimated for this model provided biologically meaningful quantities that allowed, for example, to compare the tendency of the bold fish to leave cover alone when compared to the shy fish (a measure of leadership), or the tendency of either fish to join their partner out of cover (a measure of followership). Here, we refine this characterisation of pair state to distinguish between distinct states based not only on the current location of each fish (covered or exposed) but also on the identity of the individual that initiated the most recent trip out of cover (bold fish or shy fish), and whether or not it has yet been joined by its partner (i.e. it was successful at leading). The state of a pair is thus defined by four binary variables: the location of the bold fish and the location of the shy fish (together referred to as ‘location variables’), and the identity of the most recent initiator and whether its partner has yet joined it in leaving cover (together referred to as ‘status variables’). Since not all combinations of these variables are possible, there are only 12 feasible states (numbered 1 to 12), illustrated in Figure 2. The rates (transition intensities) of all possible transitions among these 12 states were estimated by fitting the continuous-time Markov model shown in the figure. In the text, to refer to a given transition intensity, we use the notation *q_i_*_,*j*_, where *i* represents the old state and *j* the new state (thus, *q*_1,5_ is the transition intensity from state 1 to state 5). These rates provide a tendency of each individual's movement from one state to another in response to both the position of its partner and interactions (i.e. who initiated the most recent trip and whether or not it has yet been joined by its partner). Confidence intervals for transitions intensities and for their ratios (used to compare the magnitude of different transitions) were estimated by bootstrapping (1000 runs).

To test whether the status variables affected the response of a fish to its partner, we fitted a simplified model in which transition intensities were constrained such that only the two location variables mattered (i.e. a model identical to that used by Harcourt *et al.* [9], see Figure S1). The full model (including all location and status variables) was compared to the simplified model using a log-likelihood ratio test. To test whether a leader held its status within a trip, we considered an alternative, ‘one-step memory’ model in which we distinguished among eight distinct pair states, based on the current values of the location variables and on the values they took prior to the most recent state transition (see Figure S1). In this alternative model, the effects of leading or following are constrained to persist over no more than one change in location. Since the full model and the ‘one-step memory’ model are not nested, we compared the fit of these two models using the Akaike Information Criterion (AIC). To investigate in more detail how the effects of social role interact with an individual's temperament, the temperament scores of each bold and shy fish when in isolation were fitted as covariates of the full model. The log-linear effects of covariates were estimated on each transition (*β* for bold fish, *σ* for shy fish) to investigate how the temperament scores of each individual influenced the transition intensities. To refer to the covariate for a particular transition, we use subscripts with the same convention adopted for *q* (for example, *β*_1,5_ gives the effect of the temperament score of the bold fish on the transition from state 1 to state 5).

## Results

### Temperament and Coordination in Foraging Pairs

When they could observe one another's movements through a transparent partition, each fish made 53.9±3.7 (mean ± SE) trips out of and back into cover per hour. The Markov model showed that the bold fish in a pair generally had a greater propensity to leave cover than did the shy fish [(*q*_1,5_, *q*_2,5_, *q*_3,5_, *q*_4,5_)>(*q*_1,9_, *q*_2,9_, *q*_3,9_, *q*_4,9_), *P*<0.001, except *q*_2,5_ = *q*_2,9_, *P* = 0.258, *q*_2,5_ = *q*_3,9_, *P* = 0.199, *q*_2,5_<*q*_4,9_, *P*<0.001, *q_i,j_* denotes the transition intensity from state *i* to state *j*, see Figure 2] and a lesser propensity to return than did the shy fish [(*q*_6,7_, *q*_10,11_)<(*q*_6,8_, *q*_10,12_), *P*<0.001]. Focusing on the influence of the location variables, however, both the bold and the shy fish in a pair were more likely to leave cover when their partner was already out than when it was under cover [bold fish (*q*_7,6_, *q*_9,10_)>(*q*_1,5_, *q*_2,5_, *q*_3,5_, *q*_4,5_), *P*≤0.020; shy fish (*q*_5,6_, *q*_8,6_, *q*_11,10_)>(*q*_1,9_, *q*_2,9_, *q*_3,9_, *q*_4,9_), *P*<0.001]. Also, both fish were less likely to return to cover when their partner was still out than when their partner was under cover [bold fish (*q*_6,7_, *q*_10,12_)<(*q*_5,1_, *q*_8,3_, *q*_11,4_) *P*<0.001; shy fish (*q*_6,8_, *q*_10,11_)<(*q*_7,3_, *q*_9,2_, *q*_12,4_), *P*<0.001].

### Evidence for Social Roles within a Trip

The full model, distinguishing between social roles based on the memory of which fish led out of cover, gave a significant improvement in fit compared to the constrained, ‘memory-free’ version (*χ* ^2^_16_ = 731.7, *P*<0.001; ΔAIC = 699.7). Also, the full model yielded a better fit than a partially constrained model in which transition intensities depend only on the identity of the most recent initiator, but are independent of whether it has yet been joined by its partner (*χ* ^2^_8_ = 261.8, *P*<0.001; ΔAIC = 245.8). The full model showed that both members of a pair displayed greater reluctance to remain out of cover when it was their partner who first led the way, but were more resolute when they themselves were the original initiator (for the bold fish: *q*_10,12_>*q*_6,7_, *P*<0.001; for the shy fish: *q*_6,8_>*q*_10,11_, *P*<0.001). Moreover, individuals were more likely to accede to the termination of a joint trip by their partners and follow them back to cover when it was the partner who had initiated the trip (for bold: *q*_11,4_>*q*_8,3_, *P* = 0.002; for shy: *q*_7,3_>*q*_12,4_, *P*<0.001). The full model, which allows for longer-term memory of social roles, also yielded a significantly better fit than the one-step memory alternative, in which the effects of leading or following are constrained to persist over no more than one change in location (ΔAIC = 251.9).

### Exchange of Roles between Trips

Moving on from within-trip effects to between-trip effects, we see that both members of a pair were more likely to initiate a new trip out of cover if they had initiated the previous trip and successfully recruited their partner as a follower, than if it was their partner who had initiated the previous joint trip (bold fish *q*_3,5_>*q*_4,5_, *P*<0.001; shy fish *q*_4,9_>*q*_3,9_, *P*<0.001). Consequently, if we consider the sequence of initiation events (in which one individual first leaves cover after both fish have returned to safety), we see that individuals tend to have “runs" of consecutive initiations (16 out of 20 pairs have negative scores, i.e. non-random clumping, in a runs-test; binomial test: *P* = 0.012).

Shy individuals who initiated a trip out of cover but failed to recruit their bold partner as a follower were subsequently less likely to initiate the next trip compared to when they were successful at recruiting (*q*_2,9_<*q*_4,9_, *P*<0.001). On the other hand, bold individuals were insensitive to failure in recruiting a follower (*q*_1,5_ = *q*_3,5_, *P* = 0.176).

### Interaction between Social Roles and Individual Temperament

Including temperament scores of both the bold and the shy individuals as covariates in the model led to a significant improvement in fit (*χ* ^2^_48_ = 1085.8, *P*<0.001; ΔAIC = 989.8), and was superior to a model with the temperament score of only one individual included as a covariate (bold temperament alone *χ* ^2^_24_ = 364.5, *P*<0.001; ΔAIC = 316.5; shy temperament alone *χ* ^2^_24_ = 839.4, *P*<0.001; ΔAIC = 791.4). Moreover, when the effects of covariates were constrained so that an individual's temperament affected its own transition intensities but not its partner's (i.e. the effects of an individual's temperament score on the transition intensities of its partner were fixed at zero), there was a significant decrease in the model's explanatory power (*χ* ^2^_24_ = 362.0, *P*<0.001; ΔAIC = 314.0).

**Figure 3:**
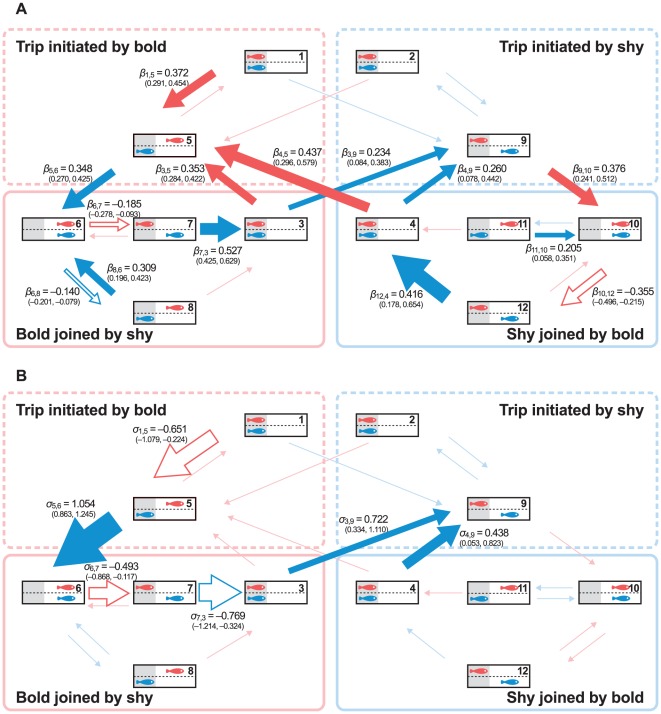
Log-linear effects of individual temperament scores. Log-linear effects of temperament scores (best estimates and 95% CI) of (A) bold individuals, and (B) shy individuals, on transition intensities. Red arrows indicate the effects of temperament scores on movements of bold individuals, and blue arrows are the effect of temperament scores on movements of shy individuals. Filled arrows: positive effects, open arrows: negative effects, arrows with lighter colour: non-significant effects. The width of each arrow indicates the magnitude of the log-linear effect.

As shown in Figure 3, bold individuals with higher temperament scores were more likely to be followed by their partner (95% CI: 0.270<*β*_5,6_<0.425, 0.425<*β*_7,3_<0.629, 0.178<*β*_12,4_<0.654, where *β_i_*_,*j*_ denotes the log-linear effect of the temperament score of the bold individual on the transition intensity from state *i* to state *j*). Also, bold individuals with higher temperament scores were more likely to ignore their partner's attempts to terminate a joint trip (0.196<*β*_8,6_<0.423, 0.058<*β*_11,10_<0.351). Finally, bold individuals whose partners had low temperament scores were more likely to initiate trips (−1.079<*σ*_1,5_<−0.224, *σ_i_*_,*j*_ denotes a log-linear effect of a temperament score of the shy individual from state *i* to state *j*).

During trips initiated by their partner, shy individuals were less likely to return to cover if their partner had a high temperament score (−0.201<*β*_6,8_<−0.079), but no such effect was present during trips that they had initiated themselves (*β*_10,11_ = 0; *P*>0.05). On the other hand, shy fish were always more likely to follow their partner back to cover if the latter had a high temperament score, irrespective of who initiated the trip (0.425<*β*_7,3_<0.629; 0.178<*β*_12,4_<0.654). Bold fish showed a reduced tendency to return to cover if paired with individuals with low temperament scores only during trips they had initiated themselves (−0.868<*σ*_6,7_<−0.117), but not during trips initiated by their partner (*σ*_10,12_ = 0; *P*>0.05).

## Discussion

Our results show that the bold fish in a pair generally had a greater propensity to leave cover than did the shy fish, confirming that individual variation in temperament is expressed consistently between asocial and social contexts (as also shown by [15]–[20]), and can be a predictor of leadership [17], [21]–[24]. Moreover, our results indicate that the fish do not only respond to the current location of their partner, but also adopt different roles depending on which individual initiated the most recent emergence from cover, and whether or not its partner has yet joined it (Figure 2). Both members of a pair become more responsive to their partner's behaviour when the latter has taken on the role of leader, and less responsive when they led the way out of cover themselves. An individual's status as leader of the most recent trip out of cover can be retained over multiple changes in location within that trip (0–18 per joint trip, with mean 0.8 ± 0.1), and social roles are reset only when both individuals return to safety and the former follower initiates a new trip out of cover.

Our results also show how pairs exchange social roles between trips. Both members of a pair were more likely to initiate a new trip out of cover if they had initiated the previous trip and successfully recruited their partner as a follower, than they were if it was their partner who had initiated the previous joint trip. Successful individuals tend to initiate more trips, and this gives them even more chances to be a successful initiator, exaggerating any underlying difference in temperament. This pattern may help to explain the finding of Harcourt *et al.* [9] that differences in temperament between members of a pair are reinforced by positive social feedback. There was also an influence of relative temperament on an individual's response to success or failure in recruiting their partner as a follower when they led a trip out of cover. Shy individuals were less likely to initiate the next trip when they failed to recruit their bold partner as a follower in the most recent trip, whereas bold individuals were insensitive to failure in recruiting a follower. Again, this effect may help to explain the finding of Harcourt *et al.* [9] that bold individuals were less sensitive to a partner's presence and behaviour than were shy individuals, as also found in other studies [13], [16], [19], [20], [25], [26].

Models of coordinated movement generally focus on the maintenance of cohesion while groups are on the move [27]–[30]; within moving shoals, cohesion arises from local responses to neighbours, with individuals in the front exerting stronger influence [31]–[33] (but see [34]). In our experiments, where we tracked pairs of individuals over multiple movements, it is clear that the act of initiating a joint trip confers greater influence on individuals' future movements, and that this effect can persist through multiple changes in position, until both pair members return to the safety of cover (the full model yielded a significantly better fit than the ‘memory-free’ model and the ‘one-step memory’ model). In other words, influence is not only a consequence of current position, but depends on an individual's role (during a particular joint trip) as initiator or follower, which in turn depends on which pair member was the first to leave cover.

Temperamental differences exert a persistent influence on behaviour, but leadership changes dynamically on a much shorter time-scale, with individuals altering their responses to one another as they exchange roles between trips. Such alterations in behaviour are not attributable in this case to changes in energy reserves (as suggested in [8], [14]), because no food was supplied during the experiments, and because there were negligible changes in the energy reserves of the fish over the short observation period of our study. In addition, fish were all of similar size (45±5 mm in standard length), and had identical feeding histories over the pre-trial period, minimising possible differences in energy budget (see Materials and Methods). More generally, such alterations cannot be explained simply by fluctuations in any aspect of an individual's motivational state (e.g. periods of higher or lower activity), because they depend upon success or failure in recruiting a partner during joint trips. Moreover, failure to recruit a partner influenced the subsequent behaviour of individuals differently depending on their temperaments.

Many previous studies have reported short-term changes in the identity of the leader between trips within animal groups [35]–[39]. None that we are aware of, however, have shown that these shifts in social role between trips are associated with changes in the ways that individuals respond to one another. Harcourt *et al.* [11] conducted an experiment similar to the one described here, but in which the fish were offered a choice between two alternative, exposed foraging sites, and members of a pair were trained to expect food at opposite locations. They found that pairs tended to take turns together visiting first one site and then the other, and that individuals were more likely to lead joint trips in their preferred direction, during which they were less likely to follow their partner back to cover. The latter finding is analogous to our observation that individuals were less likely to follow their partner to cover during trips that had initiated themselves. In our experiment there was no conflict in the direction of movement, but individuals can still disagree on the timing of leaving cover and returning to cover. Thus, individuals that take the initiative to lead a joint trip, irrespective of the type of conflict, become less responsive to their partners.

Recent studies discuss the relationship between initiative and leadership in coordinated group movement [40]–[42]. Our present results show that, in pairs of foraging sticklebacks, leadership is not simply the outcome of pre-existing individual differences in temperament. Rather, individuals actively gain influence (at least in the short term) by taking the initiative in group movement, just as they can lose influence by hanging back. Furthermore, success in recruiting a partner has an effect in determining an individual's propensity to initiate future trips, at least for the shyer member of the pair. Recent work on collective movement in larger groups has shown that interactions between nearest neighbours are sufficient to explain synchronisation of direction and speed [32], [33]; it will be interesting to see whether the lasting effects of initiation described in this paper scale up to larger groups when multiple trips are considered. Given the number of animal social systems in which there are reports of short-term changes in leadership between successive group movement [35]–[39], there is a wealth of opportunities to further explore how initiative and personality interact to determine leadership. We hope, therefore, to encourage future investigation of how individuals respond to changes in their social role, and how the behaviour of leaders contributes to their dominance within the group.

## Supporting Information

Appendix S1 **Animal Collection and Maintenance.**
(DOCX)

Figure S1 **Schematic diagrams of alternative Markov chain models.**
(DOCX)
