# Supplementary material for: Initiative, Personality and Leadership in Pairs of Foraging Fish
Source: PLoS One. 2012 May 2;7(5):e36606. doi: 10.1371/journal.pone.0036606 (PMC3342251; doi:10.1371/journal.pone.0036606)
Supplement: Appendix S1 — Animal Collection and Maintenance. (DOCX) [file pone.0036606.s001.docx]

**Appendix S1**

Animal Collection and Maintenance

Wild three-spined sticklebacks (*Gasterosteus aculeatus*) were collected from a tributary of the River Cam, about 10 km northeast of Cambridge, UK, in 2006. Fish were kept in large glass aquaria with plastic plants and under-gravel filtration systems, and allowed to acclimate to laboratory conditions for at least one month before running the experiments. During this period, fish were fed frozen bloodworms (Chironomid larvae) *ad libitum* daily. Water temperature was kept at 17 ± 1°C, and the light-cycle was set at 10-h light / 14-h dark. Fish were not sexed, as the temperature and photocycle prevented them from attaining sexual maturation [43]. Prior to the experiment, fish were transferred to holding tanks (60 × 30 × 40 cm) with under-gravel filtration systems, each of which was divided into six compartments with transparent plastic partitions. Fish were kept individually in each compartment for identification. Each compartment contained a plastic plant at one end and a white square plastic plate (about 1.5 cm^2^) at the other end as a feeding site. Fish of similar sizes (45 ± 5 mm in standard length) were selected to prevent size effects. Animal care and the experiment procedures were approved by the Animal Users Management Committee of the University of Cambridge under a non-regulated procedures regime.
