# Supplementary material for: Initiative, Personality and Leadership in Pairs of Foraging Fish
Source: PLoS One. 2012 May 2;7(5):e36606. doi: 10.1371/journal.pone.0036606 (PMC3342251; doi:10.1371/journal.pone.0036606)
Supplement: Figure S1 — Schematic diagrams of alternative Markov chain models. (DOCX) [file pone.0036606.s002.docx]

**Supplementary Figure S1**

**Supporting Information Legend**

**Figure S1. Schematic diagrams of alternative Markov chain models.**

In the ‘simplified’ model (A), state transitions are constrained to take equal intensities regardless of who initiated or whether it has yet been joined by its partner, resulting in 8 transitions between 4 states simply based on the positions of two fish. In the ‘one-step memory’ model (B), the simplified model is expanded by adding the memory of the previous transition, resulting in 16 transitions between 8 states. Here, the same positions of the two fish are distinguished depending on which state they were from. For each state, the area under cover is shaded, while the exposed area is in white. Colour of arrows corresponds to the movement of bold fish (red) and shy fish (blue).
